# Supplementary material for: MLL oncoprotein levels influence leukemia lineage identities
Source: Nat Commun. 2024 Oct 29;15:9341. doi: 10.1038/s41467-024-53399-8 (PMC11522475; doi:10.1038/s41467-024-53399-8)
Supplement: Supplementary file 1 — Supplementary Information [file 41467_2024_53399_MOESM1_ESM.pdf]

Supplementary Information

MLL oncoprotein levels influence leukemia lineage identity

D. H. Janssens *et* al.

This PDF file includes Supplementary Figures 1 to 5

Supplementary Fig 1: MLL N-terminal signal is enriched over canonical oncoprotein-target genes.

Supplementary Fig 2: Comparisons of MLL-oncoprotein scores and identification of minimal MLL-fusion-partner exon junctions.

Supplementary Fig 3: MLL oncoproteins have heterogeneous expression levels that result in differential oncoprotein-target gene regulation.

Supplementary Fig 4: Principal Components 1 and 2 capture lineage-specific and fusion-partner-dependent oncoprotein-target genes.

Supplementary Fig 5: Identification of B-ALL and AML specific oncoprotein-target genes during lineage switching.

Supplementary Fig 6: Loss of oncoprotein binding and altered target-gene expression in the menin inhibitor treated lineage-switching sample.

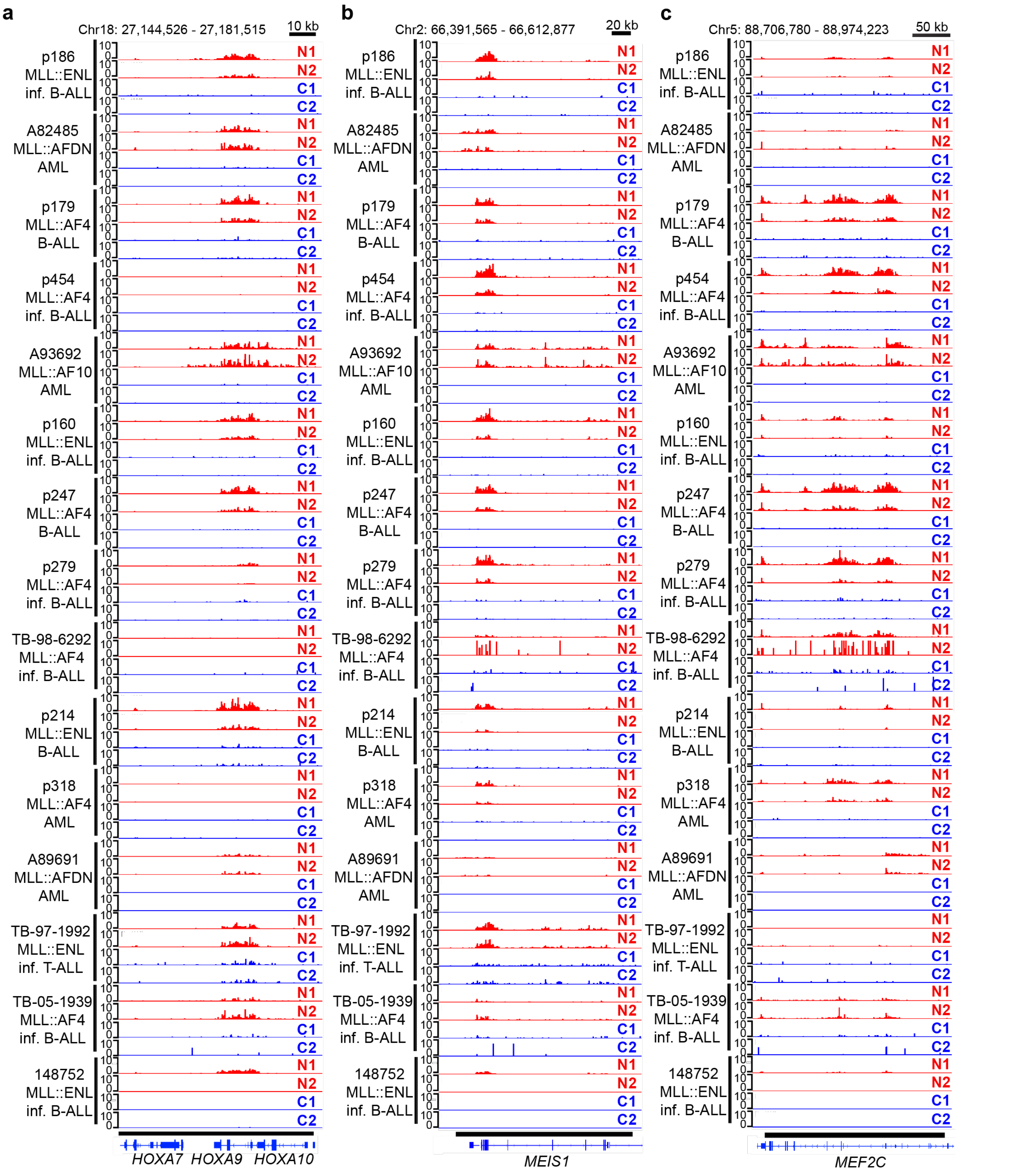

Supplementary Fig 1: MLL N-terminal signal is enriched over canonical oncoprotein-target genes. a, Genome Browser tracks of the *HOXA* locus show the generally consistent profiles between the two MLL N-terminal antibodies (red; N1 and N2) and the two MLL C-terminal antibodies (blue; C1 and C2) signal in fifteen of the *MLL*-rearranged leukemias; bottom black bar = oncoprotein-target peak. **b,** Same as (a), but showing the signal over the *MEIS1* locus. The signal spikes seen in a small number of tracks (e.g. TB-98-6292 N2) are indicative of over sequencing and an increased proportion of PCR duplicates. For all downstream analysis, PCR duplicates were removed from all samples prior to combining the N1 and N2 datasets as well as the C1 and C2 datasets. **c,** Same as (a), but showing the signal over the *MEF2C* locus.

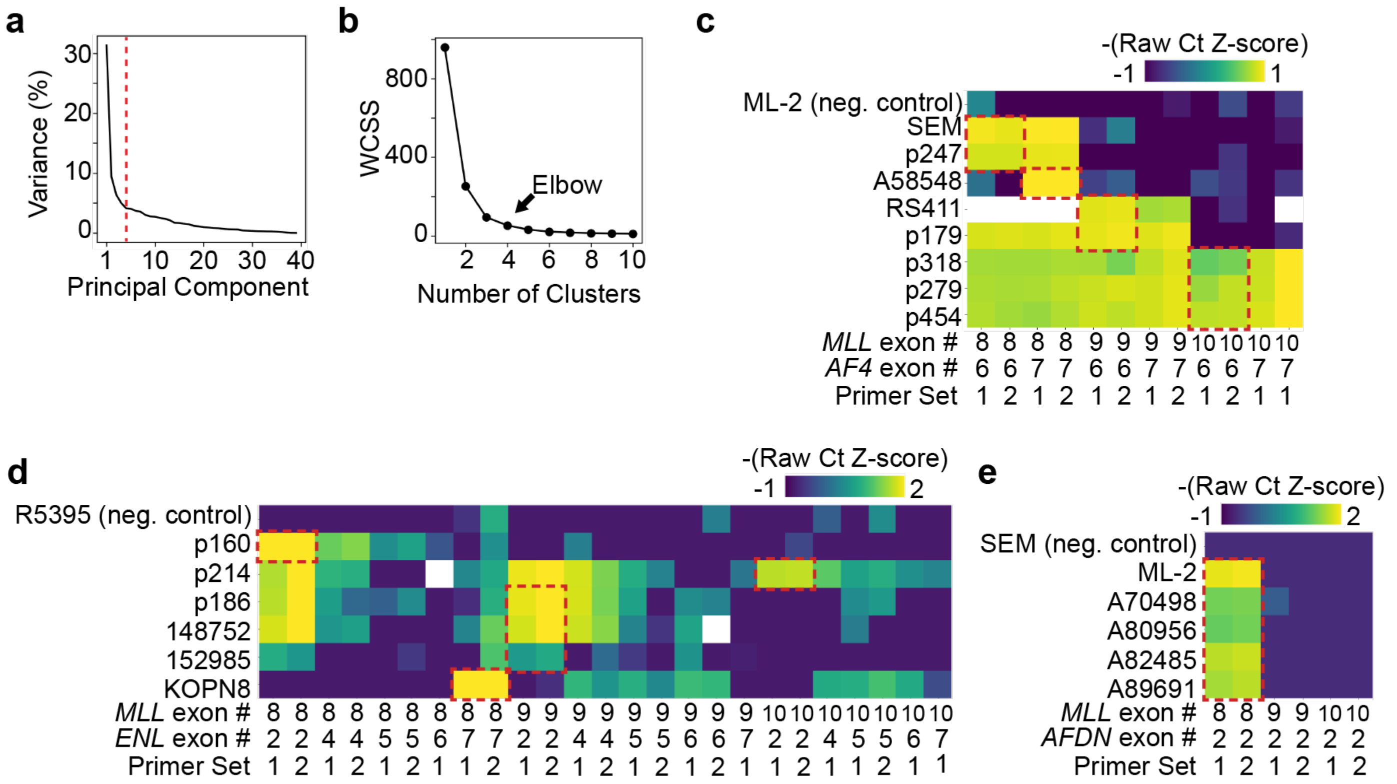

Supplementary Fig 2: Comparisons of MLL-oncoprotein scores and identification of minimal MLL-fusion-partner exon junctions. a, Rank order plot of the variance in oncoprotein scores detected by principal component analysis. The first 4 components (red line) capture >50% of the total variance. **b,** Elbow plot of the K-means Within-Cluster Sum of Squares (WCSS) scores suggests the *MLL*r leukemias form 4 clusters in UMAP space. **c,** Heatmap of the z-scaled raw crossing-threshold (ct) values for qPCR reactions targeting the exons flanking the most common breakpoints in the *MLL::AF4* translocations. Red boxes indicate the minimal MLL-fusion exon junctions. These primers were used to compare the relative oncogene expression between samples with the same minimal exon junctions in Fig. 1h. **d,** Same as (c) for *MLL::ENL* exon junctions. **e,** Same as (c) for *MLL::AFDN* exon junctions. Source data are provided as a Source Data file.

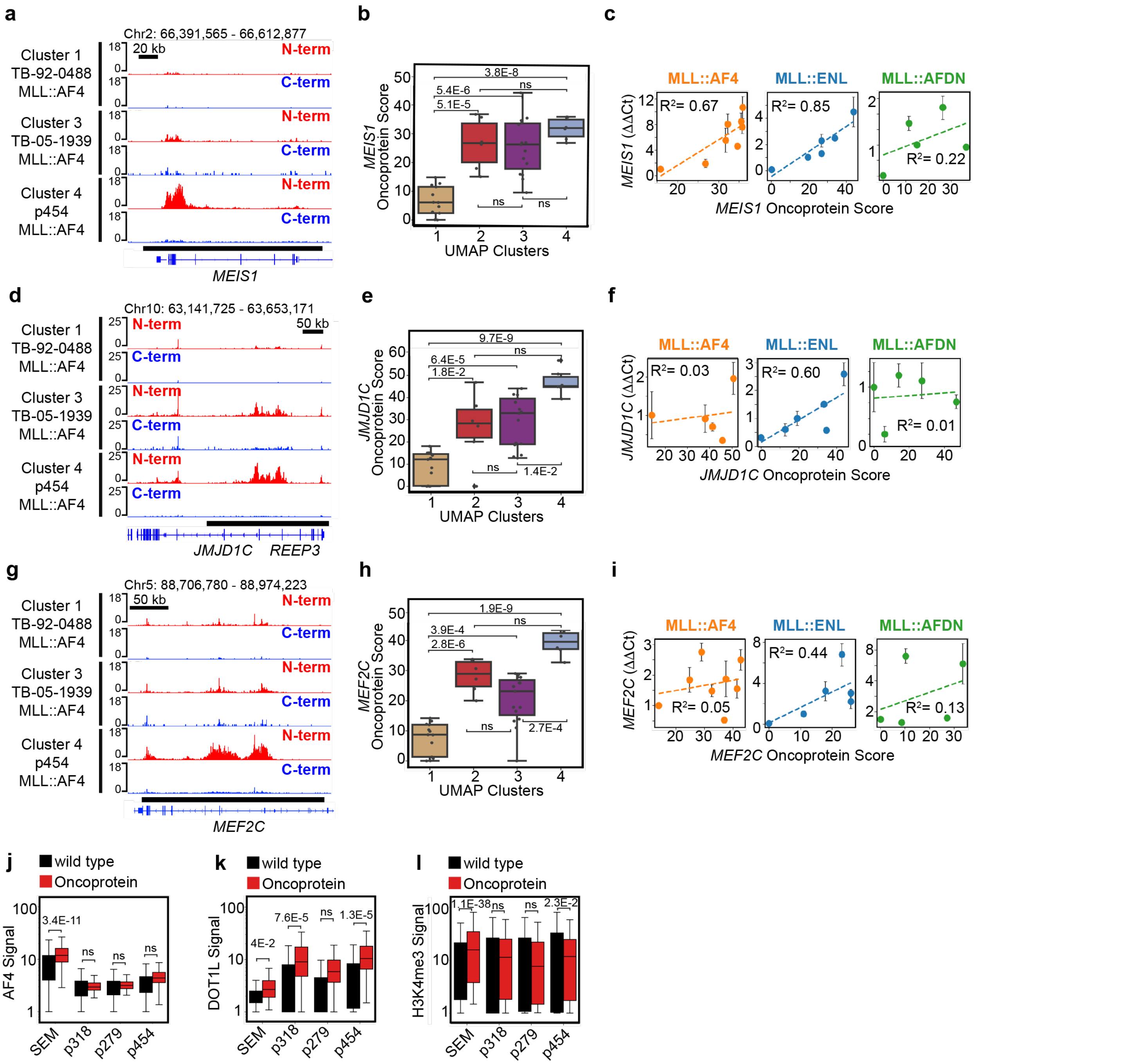

Supplementary Fig 3: MLL oncoproteins have heterogeneous expression levels that result in differential oncoprotein-target gene regulation. a, Genome Browser tracks of the *MEIS1* locus show the difference in MLL N-terminal (red) and C-terminal (blue) signal in three *MLL::AF4* rearranged leukemias; bottom black bar = oncoprotein-target peak. **b,** Boxplot comparison of the oncoprotein scores over *MEIS1* split into the four UMAP clusters from Fig. 1d; *p* values were computed using a two-tailed independent samples t-test; Cluster 1 n = 13 samples, Cluster 2 n = 6 samples, Cluster 3 n = 15 samples, Cluster 4 n = 5 samples; boxplot center lines = median, box limits = first and third quartiles, whiskers = 1.5 times the interquartile range (IQR). **c,** Scatterplots showing the correlation between the gene-specific oncoprotein scores and the expression of *MEIS1*; dotted lines indicate the regression and R^2 measures the fit to the data. **d,** Same as (a) showing the *JMJD1C* locus. **e,** Same as (b) showing *JMJD1C* oncoprotein scores. **f,** Same as (c) showing *JMJD1C* oncoprotein scores and expression. **g,** Same as (a) showing the *MEF2C* locus. **h,** Same as (b) showing *MEF2C* oncoprotein scores. **i,** Same as (c) showing *MEF2C* oncoprotein scores and expression. **j,** Boxplot showing the relative enrichment of AF4 over wild-type and oncoprotein-target sites in four *MLL::AF4*-rearranged samples. *p* values were computed using a two-tailed independent samples t-test; *n* values are listed as wild-type site #, oncoprotein site #: SEM = 15611, 491; p318 = 22014, 246; p279 = 25672, 272; p454 = 18509, 438; boxplot center lines = median, box limits = first and third quartiles, whiskers = 1.5 times the interquartile range (IQR). **k,** Same as (j) but comparing the DOT1L signal intensity. **l,** Same as (j) but comparing the H3K4me3 signal intensity. Source data are provided as a Source Data file.

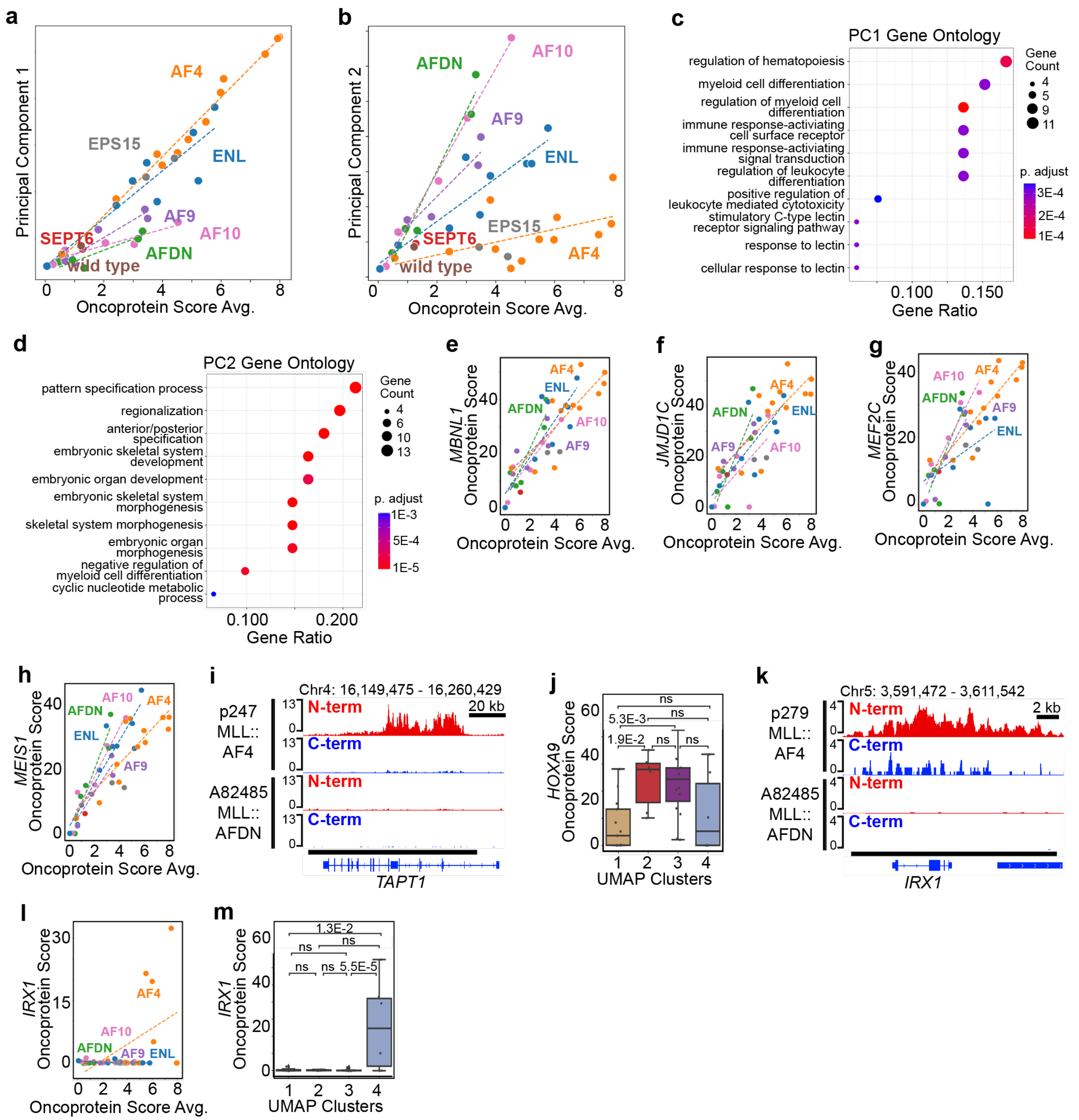

Supplementary Fig 4: Principal Components 1 and 2 capture lineage-specific and fusion-partner-dependent oncoprotein-target genes. a, Scatterplot showing the average oncoprotein scores for AF4 and ENL fusion oncoproteins are more strongly associated with Principal Component 1. **b,** The average oncoprotein scores for AFDN and AF10 fusion oncoproteins are more strongly associated with Principal Component 2. **c,** Dot plot showing the significance of the top GO terms related to the oncoprotein-target genes that contribute to Principal Component 1. **d,** Same as (c) showing the GO terms associated with Principal Component 2. **e,** Scatterplot showing the *MBNL1* oncoprotein scores relative to the average oncoprotein score in each sample. Samples are colored by the MLL-fusion partner and dotted lines indicate the regression. **f,** Same as (e), but showing the *JMJD1C* oncoprotein scores. **g,** Same as (e), but showing the *MEF2C* oncoprotein scores. **h,** Same as (e), but showing the *MEIS1* oncoprotein scores. **i,** Genome Browser tracks show the MLL::AF4 oncoprotein binds the *TAPT1* locus while the MLL::AFDN oncoprotein does not (bottom black bar = oncoprotein-target peak). **j,** Boxplot comparison of the oncoprotein scores over *HOXA9* split into the four UMAP clusters from Fig. 1d; *p* values were computed using a two-tailed independent samples t-test; Cluster 1 n = 13 samples, Cluster 2 n = 6 samples, Cluster 3 n = 15 samples, Cluster 4 n = 5 samples; boxplot center lines = median, box limits = first and third quartiles, whiskers = 1.5 times the interquartile range (IQR). **k,** Same as (i) showing the *IRX1* locus, and the *MLL::AF4*-rearranged sample from Fig. 4h in which *HOXA9* is not called as an MLL-oncoprotein-target gene. **l,** Same as (e), but showing the *IRX1* oncoprotein scores. **m,** Same as (j), but showing the *IRX1* oncoprotein scores. Source data are provided as a Source Data file.

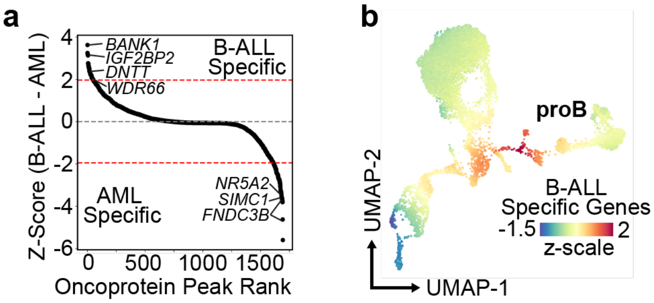

Supplementary Fig 5: Identification of B-ALL and AML specific oncoprotein-target genes during lineage switching. a, Rank order plot of the difference in oncoprotein scores between the B-ALL and AML patient matched sample. A threshold of the top 5% and bottom 5% of sites was used to call B-ALL-specific and AML-specific oncoprotein-target sites, respectively. **b,** The UMAP embedding of healthy lineage-depleted bone marrow colored by the average z-scores of the 66 B-ALL-specific oncoprotein target genes from Fig. 5g.

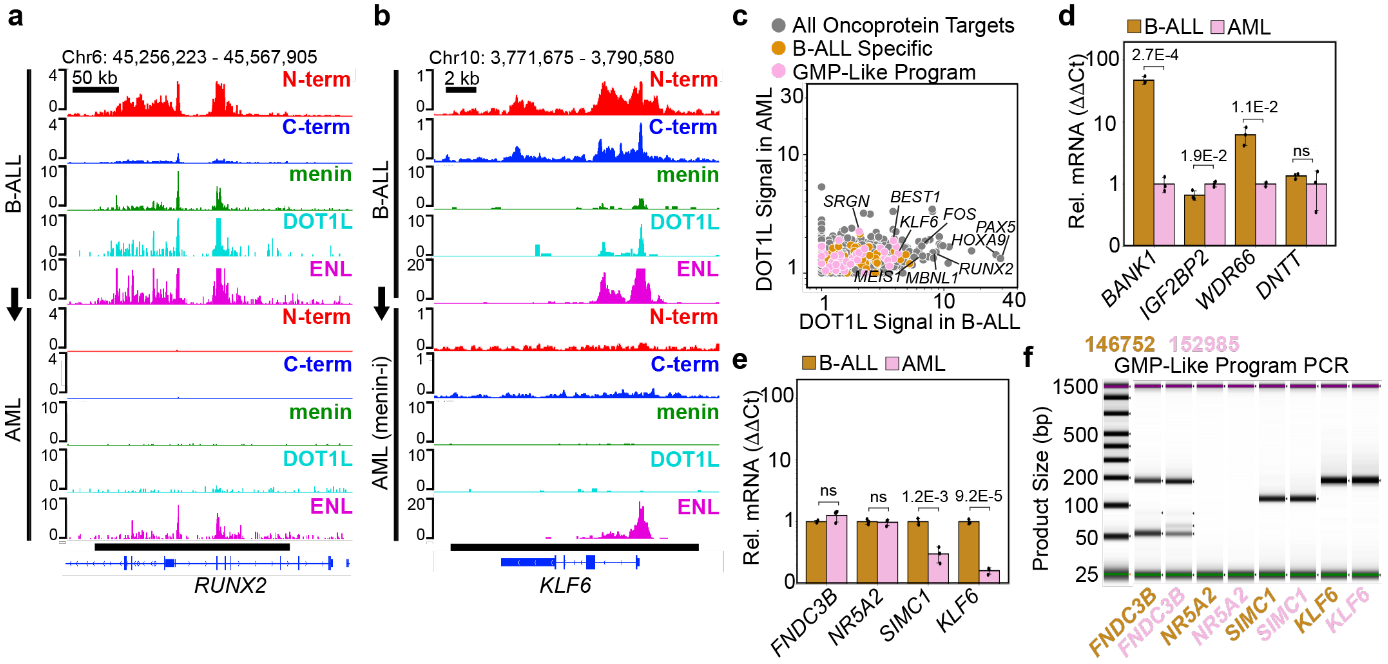

Supplementary Fig 6: Loss of oncoprotein binding and altered target-gene expression in the menin inhibitor treated lineage-switching sample. a, Genome Browser tracks showing the oncoprotein and the cofactors menin, DOT1L and ENL bound to the *RUNX2* locus in the B-ALL sample (top). In the patient-matched AML sample treated with the menin inhibitor, only ENL remains bound (bottom). **b,** Same as (a) but showing the *KLF6* gene from the GMP-like program. **c,** Scatterplot comparing the normalized DOT1L levels in the B-ALL and the AML sample treated with the menin inhibitor over an internal control group of oncoprotein-target sites (grey) the B-ALL-specific program (gold) and the GMP-like program (pink). **d,** The B-ALL-specific oncoprotein target genes *BANK1* and *WDR66* are expressed at significantly higher levels in the B-ALL sample (gold) prior to lineage switching than the AML sample (pink). Bar height is the average of three qPCR biological replicates; Error bars = standard deviation; p value computed using a two-tailed independent samples t-test; $n = 3$ qPCR biological replicates. **e,** Same as (d) but showing the relative expression of genes in the GMP-like program. Expression of *SIMC1* and *KLF6* is reduced in the menin-inhibitor-treated AML sample. **f,** Real time PCR indicates *FNDC3B*, *SIMC1* and *KLF6* are expressed in the B-ALL and AML menin-inhibitor-treated lineage-switching samples and that *NR5A2* is not expressed. Source data are provided as a Source Data file.
