# Supplementary material for: MLL oncoprotein levels influence leukemia lineage identities
Source: Nat Commun. 2024 Oct 29;15:9341. doi: 10.1038/s41467-024-53399-8 (PMC11522475; doi:10.1038/s41467-024-53399-8)
Supplement: Supplementary file 2 — Description of Additional Supplementary Files [file 41467_2024_53399_MOESM2_ESM.pdf]

Description of Additional Supplementary Files

MLL oncoprotein levels influence leukemia lineage identity

D. H. Janssens *et* al.

Supplementary Data 1: *MLL*r leukemia sample descriptions.

Supplementary Data 2: Table of MLL oncoprotein target genes called in each sample.

Supplementary Data 3: Table of top oncoprotein peaks and genes that contribute to PC1 and PC2.

Supplementary Data 4: Table of lineage-switching B-ALL and AML-specific oncoprotein target genes.

Supplementary Data 5: Table of qPCR primer sequences.
