# Supplementary material for: MLL oncoprotein levels influence leukemia lineage identities
Source: Nat Commun. 2024 Oct 29;15:9341. doi: 10.1038/s41467-024-53399-8 (PMC11522475; doi:10.1038/s41467-024-53399-8)
Supplement: Supplementary file 8 — Reporting Summary [file 41467_2024_53399_MOESM8_ESM.pdf]

nature research

Corresponding author(s): Steven Henikoff

Last updated by author(s): September 21 2024

Reporting Summary

Nature Research wishes to improve the reproducibility of the work that we publish. This form provides structure for consistency and transparency in reporting. For further information on Nature Research policies, see our Editorial Policies and the Editorial Policy Checklist.

Statistics

For all statistical analyses, confirm that the following items are present in the figure legend, table legend, main text, or Methods section.

n/a	Confirmed	
☐	☒	The exact sample size (*n*) for each experimental group/condition, given as a discrete number and unit of measurement
☐	☒	A statement on whether measurements were taken from distinct samples or whether the same sample was measured repeatedly
☐	☒	The statistical test(s) used AND whether they are one- or two-sided<br>*Only common tests should be described solely by name; describe more complex techniques in the Methods section.*
☒	☐	A description of all covariates tested
☐	☒	A description of any assumptions or corrections, such as tests of normality and adjustment for multiple comparisons
☐	☒	A full description of the statistical parameters including central tendency (e.g. means) or other basic estimates (e.g. regression coefficient) AND variation (e.g. standard deviation) or associated estimates of uncertainty (e.g. confidence intervals)
☐	☒	For null hypothesis testing, the test statistic (e.g. *F*, *t*, *r*) with confidence intervals, effect sizes, degrees of freedom and *P* value noted<br>*Give P values as exact values whenever suitable.*
☒	☐	For Bayesian analysis, information on the choice of priors and Markov chain Monte Carlo settings
☒	☐	For hierarchical and complex designs, identification of the appropriate level for tests and full reporting of outcomes
☐	☒	Estimates of effect sizes (e.g. Cohen's *d*, Pearson's *r*), indicating how they were calculated

Our web collection on statistics for biologists contains articles on many of the points above.

Software and code

Policy information about availability of computer code

Data collection: The size distributions and molar concentration of libraries were determined using an Agilent 4200 TapeStation. Up to 48 barcoded CUT&RUN libraries were pooled at approximately equimolar concentration for sequencing. Paired-end 50×50 bp sequencing on the Illumina NextSeq 2000 platform was performed by the Fred Hutchinson Cancer Center Genomics Shared Resources. This yielded 5-10 million reads per antibody. To remove adapter sequences, we preprocessed the reads using cutadapt version 2.9 with parameters -j 8 --nextseq-trim 20 -m 20 -a AGATCGGAAGAGCACACGTCTGAACTCCAGTCA -A AGATCGGAAGAGCGTCGTGTAGGGAAAGAGTGT -Z. Paired-end reads were then aligned to the UCSC hg38 human genome build using Bowtie2 version 2.4.4 with parameters --very-sensitive-local --soft-clipped-unmapped-tlen --dovetail --no-mixed --no-discordant -q --phred33 -I 10 -X 1000.

Data analysis: Bedtools; Deeptools; Python Packages Used: Numpy, Pandas, Seaborn, Matplotlib, umap, SciPy; R version 4.0.0, R libraries used: MASS, ggplot2, Rtsne, densityClust, gplots, heatmap3, RColorBrewer; Custom Code will be provided during review

For manuscripts utilizing custom algorithms or software that are central to the research but not yet described in published literature, software must be made available to editors and reviewers. We strongly encourage code deposition in a community repository (e.g. GitHub). See the Nature Research guidelines for submitting code & software for further information.

Data

Policy information about availability of data

All manuscripts must include a data availability statement. This statement should provide the following information, where applicable:
- Accession codes, unique identifiers, or web links for publicly available datasets
- A list of figures that have associated raw data
- A description of any restrictions on data availability

The CUT&RUN and CUT&Tag sequencing data that was generated for this study has been deposited into the Gene Expression Omnibus and is available under the

accession code GSE252378 (https://www.ncbi.nlm.nih.gov/geo/query/acc.cgi?acc=GSE252378). For seven of the patient samples we profiled (p160, p179, p186, p214, p247, p279 and p318) the raw sequencing data are not publicly available and will not be shared because patients did not consent to genomic data sharing. For these samples the aligned bed and bigwig files are available as part of the GSE252378 dataset, and these bed files are also available through Zenodo48 DOI 10.5281/zenodo.13791761. The raw sequencing data for all the other samples described in this study is publicly available. The additional CUT&RUN data analyzed in this study was generated by Janssens et al. 202119 and the raw paired-end fastq files are available through the Gene Expression Omnibus under accession code GSE159608 (https://www.ncbi.nlm.nih.gov/geo/query/acc.cgi?acc=GSE159608). Source data are provided with this paper. The remaining data are available within the Article, Supplementary Information or Source Data file.

Field-specific reporting

Please select the one below that is the best fit for your research. If you are not sure, read the appropriate sections before making your selection.

- [x] Life sciences
- [ ] Behavioural & social sciences
- [ ] Ecological, evolutionary & environmental sciences

For a reference copy of the document with all sections, see nature.com/documents/nr-reporting-summary-flat.pdf

Life sciences study design

All studies must disclose on these points even when the disclosure is negative.

Sample size	In this study we collected chromatin profiling data for comparative analysis from 24 KMT2Ar primary patient leukemia samples. Each sample was profiled using two antibodies targeting the N terminus and 2 antibodies targeting the C terminus. We also collected CUT&RUN data using two antibodies targeting the AF4 KMT2A-oncoprotein-fusion partner. For the AutoCUT&Tag profiling of the oncoprotein transcriptional cofactors menin, DOT1L and ENL as well as H3K4me3 we collected two biological replicates.
Data exclusions	Sequencing reads mapping to the mitochondrial genome were removed from all datasets. This was pre-established and is standard practice in the field. The purpose of this study was to perform comparative analysis of chromatin profiles from the nuclear genome and this can be confounded by variable read numbers from the mitochondrial genome.
Replication	At least 2 biological replicates were profiled. All attempts at replication were successful.
Randomization	n/a. The data and analysis for this study is objective and not prone to influence by the researchers bias.
Blinding	n/a. The data and analysis for this study is objective and not prone to influence by researchers bias.

Reporting for specific materials, systems and methods

We require information from authors about some types of materials, experimental systems and methods used in many studies. Here, indicate whether each material, system or method listed is relevant to your study. If you are not sure if a list item applies to your research, read the appropriate section before selecting a response.

Materials & experimental systems

n/a	Involved in the study	
[ ]	[x]	Antibodies
[x]	[ ]	Eukaryotic cell lines
[x]	[ ]	Palaeontology and archaeology
[x]	[ ]	Animals and other organisms
[ ]	[x]	Human research participants
[x]	[ ]	Clinical data
[x]	[ ]	Dual use research of concern

Methods

n/a	Involved in the study	
[ ]	[x]	ChIP-seq
[x]	[ ]	Flow cytometry
[x]	[ ]	MRI-based neuroimaging

Antibodies

Antibodies used

mouse monoclonal anti-KMT2A (1:100, Millipore, clone N4.4, Cat# 05-764),
rabbit monoclonal anti-KMT2A (1:100, Cell Signaling Tech, clone D2M7U, Cat# 14689S),
mouse monoclonal anti-KMT2A (1:100, Millipore, clone 9-12, Cat# 05-765),
mouse monoconal anti-KMT2A (1:100, Santa Cruz, clone H-10, Cat# sc-374392),
rabbit anti-Mouse IgG (1:100, Abcam Cat# ab46540)
mouse monoclonal anti-AF4 (1:50, MyBioSource Cat# MBS190886)
rabbit polyclonal anti-AF4 (1:50, Thermo Fischer Scientific Cat# PA5-77068)
rabbit anti menin (1:50, Bethyl Cat# A300-105A)
rabbit anti DOT1L (1:50, Cell Signaling Tech Cat# 90878S)
rabbit anti ENL (1:50, Cell Signaling Tech Cat# 14893S)
rabbit anti H3K4me3 (1:50, Active Motif Cat# 39159)
Guinea Pig anti-Rabbit IgG (1:100, antibodies-online Cat# ABIN101961)

Validation	All antibodies are commercially available, and have been verified by Western blotting or by peptide ELISA described on the manufacturer's specification sheets. All antibodies used in this study are confirmed to recognize the human protein as stated on the manufactures website.

Human research participants

Policy information about studies involving human research participants

Population characteristics	n/a
Recruitment	n/a
Ethics oversight	n/a

Note that full information on the approval of the study protocol must also be provided in the manuscript.

ChIP-seq

Data deposition

- [x] Confirm that both raw and final processed data have been deposited in a public database such as GEO.
- [x] Confirm that you have deposited or provided access to graph files (e.g. BED files) for the called peaks.

Data access links<br>*May remain private before publication.*	*For "Initial submission" or "Revised version" documents, provide reviewer access links. For your "Final submission" document, provide a link to the deposited data.*
Files in database submission	DJ_Hs_p179_IgG_230613<br>DJ_Hs_p179_KMT2A_N1_230613<br>DJ_Hs_p179_KMT2A_N2_230613<br>DJ_Hs_p179_KMT2A_C1_230613<br>DJ_Hs_p179_KMT2A_C2_230613<br>DJ_Hs_p279_IgG_230613<br>DJ_Hs_p279_KMT2A_N1_230613<br>DJ_Hs_p279_KMT2A_N2_230613<br>DJ_Hs_p279_KMT2A_C1_230613<br>DJ_Hs_p279_KMT2A_C2_230613<br>DJ_Hs_p454_IgG_230613<br>DJ_Hs_p454_KMT2A_N1_230613<br>DJ_Hs_p454_KMT2A_N2_230613<br>DJ_Hs_p454_KMT2A_C1_230613<br>DJ_Hs_p454_KMT2A_C2_230613<br>DJ_Hs_p247_IgG_230613<br>DJ_Hs_p247_KMT2A_N1_230613<br>DJ_Hs_p247_KMT2A_N2_230613<br>DJ_Hs_p247_KMT2A_C1_230613<br>DJ_Hs_p247_KMT2A_C2_230613<br>DJ_Hs_p318_IgG_230613<br>DJ_Hs_p318_KMT2A_N1_230613<br>DJ_Hs_p318_KMT2A_N2_230613<br>DJ_Hs_p318_KMT2A_C1_230613<br>DJ_Hs_p318_KMT2A_C2_230613<br>DJ_Hs_p160_IgG_230613<br>DJ_Hs_p160_KMT2A_N1_230613<br>DJ_Hs_p160_KMT2A_N2_230613<br>DJ_Hs_p160_KMT2A_C1_230613<br>DJ_Hs_p160_KMT2A_C2_230613<br>DJ_Hs_p186_IgG_230613<br>DJ_Hs_p186_KMT2A_N1_230613<br>DJ_Hs_p186_KMT2A_N2_230613<br>DJ_Hs_p186_KMT2A_C1_230613<br>DJ_Hs_p186_KMT2A_C2_230613<br>DJ_Hs_p214_IgG_230613<br>DJ_Hs_p214_KMT2A_N1_230613<br>DJ_Hs_p214_KMT2A_N2_230613<br>DJ_Hs_p214_KMT2A_C1_230613<br>DJ_Hs_p214_KMT2A_C2_230613<br>DJ_Hs_A70498_IgG_221101<br>DJ_Hs_A70498_KMT2A_N1_221101

DJ_Hs_A70498_KMT2A_N2_221101
DJ_Hs_A70498_KMT2A_C1_221101
DJ_Hs_A70498_KMT2A_C2_221101
DJ_Hs_A80956_IgG_221101
DJ_Hs_A80956_KMT2A_N1_221101
DJ_Hs_A80956_KMT2A_N2_221101
DJ_Hs_A80956_KMT2A_C1_221101
DJ_Hs_A80956_KMT2A_C2_221101
DJ_Hs_A82485_IgG_221101
DJ_Hs_A82485_KMT2A_N1_221101
DJ_Hs_A82485_KMT2A_N2_221101
DJ_Hs_A82485_KMT2A_C1_221101
DJ_Hs_A82485_KMT2A_C2_221101
DJ_Hs_A73051_IgG_221101
DJ_Hs_A73051_KMT2A_N1_221101
DJ_Hs_A73051_KMT2A_N2_221101
DJ_Hs_A73051_KMT2A_C1_221101
DJ_Hs_A73051_KMT2A_C2_221101
DJ_Hs_A93692_IgG_221101
DJ_Hs_A93692_KMT2A_N1_221101
DJ_Hs_A93692_KMT2A_N2_221101
DJ_Hs_A93692_KMT2A_C1_221101
DJ_Hs_A93692_KMT2A_C2_221101
DJ_Hs_A89691_IgG_221101
DJ_Hs_A89691_KMT2A_N1_221101
DJ_Hs_A89691_KMT2A_N2_221101
DJ_Hs_A89691_KMT2A_C1_221101
DJ_Hs_A89691_KMT2A_C2_221101
JS_Hs_032922_JS2223
JS_Hs_032922_JS2224
JS_Hs_032922_JS2225
JS_Hs_032922_JS2226
JS_Hs_032922_JS2227
JS_Hs_032922_JS2235
JS_Hs_032922_JS2236
JS_Hs_032922_JS2237
JS_Hs_032922_JS2238
JS_Hs_032922_JS2239
JS_Hs_032922_JS2151
JS_Hs_032922_JS2152
JS_Hs_032922_JS2153
JS_Hs_032922_JS2154
JS_Hs_032922_JS2155
JS_Hs_032922_JS2156
JS_Hs_032922_JS2157
JS_Hs_032922_JS2158
JS_Hs_032922_JS2159
JS_Hs_032922_JS2160
JS_Hs_032922_JS2161
JS_Hs_032922_JS2162
JS_Hs_032922_JS2163
JS_Hs_032922_JS2164
JS_Hs_032922_JS2165
JS_Hs_032922_JS2166
JS_Hs_032922_JS2167
JS_Hs_032922_JS2168
JS_Hs_032922_JS2169
JS_Hs_032922_JS2170
JS_Hs_032922_JS2171
JS_Hs_032922_JS2172
JS_Hs_032922_JS2173
JS_Hs_032922_JS2174
JS_Hs_032922_JS2175
JS_Hs_032922_JS2176
JS_Hs_032922_JS2177
JS_Hs_032922_JS2178
JS_Hs_032922_JS2179
JS_Hs_032922_JS2180

JS_Hs_032922_JS2181
JS_Hs_032922_JS2182
JS_Hs_032922_JS2183
JS_Hs_032922_JS2184
JS_Hs_032922_JS2185
JS_Hs_032922_JS2186
JS_Hs_032922_JS2187
JS_Hs_032922_JS2188
JS_Hs_032922_JS2189
JS_Hs_032922_JS2190
JS_Hs_032922_JS2191
JS_Hs_032922_JS2192
JS_Hs_032922_JS2193
JS_Hs_032922_JS2194
JS_Hs_032922_JS2195
JS_Hs_032922_JS2196
JS_Hs_032922_JS2197
JS_Hs_032922_JS2198
JS_Hs_032922_JS2199
JS_Hs_032922_JS2200
JS_Hs_032922_JS2201
JS_Hs_032922_JS2202
JS_Hs_032922_JS2203
JS_Hs_032922_JS2204
JS_Hs_032922_JS2205
JS_Hs_032922_JS2206
JS_Hs_032922_JS2207
JS_Hs_032922_JS2208
JS_Hs_032922_JS2209
JS_Hs_032922_JS2210
JS_Hs_032922_JS2211
JS_Hs_032922_JS2212
JS_Hs_032922_JS2213
JS_Hs_032922_JS2214
JS_Hs_032922_JS2215
DJ_Hs_5395_IgG_05T_040324
DJ_Hs_5395_KMT2A_N1_05T_040324
DJ_Hs_5395_KMT2A_N2_05T_040324
DJ_Hs_5395_KMT2A_C1_05T_040324
DJ_Hs_5395_KMT2A_C2_05T_040324
DJ_Hs_5652_IgG_05T_040324
DJ_Hs_5652_KMT2A_N1_05T_040324
DJ_Hs_5652_KMT2A_N2_05T_040324
DJ_Hs_5652_KMT2A_C1_05D_040324
DJ_Hs_5652_KMT2A_C2_05D_040324
DJ_Hs_SEM_AFF1_1_05T_040324
DJ_Hs_SEM_AFF1_2_05T_040324
DJ_Hs_p279_AFF1_1_05T_040324
DJ_Hs_p279_AFF1_2_05T_040324
DJ_Hs_p318_AFF1_1_05T_040324
DJ_Hs_p318_AFF1_2_05T_040324
DJ_Hs_p454_AFF1_1_05T_040324
DJ_Hs_p454_AFF1_2_05T_040324
DJ_Hs_DJ5_148752_500K_KMT2A_N1 _240416
DJ_Hs_DJ2_148752_500K_KMT2A_N2 _240416
DJ_Hs_DJ6_148752_500K_KMT2A_C1 _240416
DJ_Hs_DJ7_148752_500K_KMT2A_C2 _240416
DJ_Hs_DJ8_152985_500K_KMT2A_N1 _240416
DJ_Hs_DJ4_152985_500K_KMT2A_N2 _240416
DJ_Hs_DJ9_152985_500K_KMT2A_C1 _240416
DJ_Hs_DJ10_152985_500K_KMT2A_C2 _240416
DJ_Hs_p279_Dot1L_R1_230801
DJ_Hs_p279_Dot1L_R2_230801
DJ_Hs_p279_K4me3_R1_230801
DJ_Hs_p279_K4me3_R2_230801
DJ_Hs_p279_ENL_R1_230801
DJ_Hs_p279_ENL_R2_230801
DJ_Hs_p318_Dot1L_R1_230801

DJ_Hs_p318_Dot1L_R2_230801
DJ_Hs_p318_K4me3_R1_230801
DJ_Hs_p318_K4me3_R2_230801
DJ_Hs_p318_ENL_R1_230801
DJ_Hs_p318_ENL_R2_230801
DJ_Hs_p454_Dot1L_R1_230801
DJ_Hs_p454_Dot1L_R2_230801
DJ_Hs_p454_K4me3_R1_230801
DJ_Hs_p454_K4me3_R2_230801
DJ_Hs_p454_ENL_R1_230801
DJ_Hs_p454_ENL_R2_230801
DJ_Hs_148752_Menin_R1_041624
DJ_Hs_148752_Menin_R2_041624
DJ_Hs_148752_Dot1L_R1_041624
DJ_Hs_148752_Dot1L_R2_041624
DJ_Hs_148752_ENL_R1_041624
DJ_Hs_148752_ENL_R2_041624
DJ_Hs_152985_Menin_R1_041624
DJ_Hs_152985_Menin_R2_041624
DJ_Hs_152985_Dot1L_R1_041624
DJ_Hs_152985_Dot1L_R2_041624
DJ_Hs_152985_ENL_R1_041624
DJ_Hs_152985_ENL_R2_041624

Genome browser session
(e.g. UCSC)

http://genome.ucsc.edu/s/djanssen/Janssens_2023_Fig_3bd_ExData_4
http://genome.ucsc.edu/s/djanssen/Janssens_2023_Fig_2de
http://genome.ucsc.edu/s/djanssen/Janssens_2023_Fig_1e

Methodology

Replicates	At least 2 replicates were performed. Comparative analysis is the topic of this manuscript.
Sequencing depth	All Experiments were paired-end. Sequencing depths and sampling is reported in the manuscript.
Antibodies	All antibodies and sources are provided in the Methods section.
Peak calling parameters	SEACR version 1.3, "stringent" mode, “norm” mode. This is stated in the methods section.
Data quality	Data quality assessment is the topic of this manuscript, and is reported.
Software	The code used for processing CUT&RUN and CUT&Tag Data (e.g. removing duplicates and combining replicates) as well as the preprocessed data tables, and the python jupyter notebooks used for figure generation are available at: https://github.com/DerekJanssens/MLL_oncoprotein_levels_NatComms and are also published on Zenodo (https://doi.org/10.5281/zenodo.13791762). In jupyter we used the following packages: pandas version 1.5.3, numpy version 1.25.2, matplotlib version 3.9.2, seaborn version 0.13.2, scipy version 1.14.1, statsmodels version 1.5.3, scikit-learn version 1.25.2, umap-learn version 1.5.3, matplotlib-venn version 1.25.2
